# A Comprehensive Evaluation Method for Soil Remediation Technology Selection: Case Study of Ex Situ Thermal Desorption

**DOI:** 10.3390/ijerph19063304

**Published:** 2022-03-11

**Authors:** Shuang Li, Liao He, Bo Zhang, Yan Yan, Wentao Jiao, Ning Ding

**Affiliations:** 1 State Key Laboratory of Urban and Regional Ecology, Research Center for Eco-Environmental Sciences, Chinese Academy of Sciences, Beijing 100085, China; lishuang_1216@126.com (S.L.); 2018520089@bipt.edu.cn (L.H.); zhangbo@rcees.ac.cn (B.Z.); yyan@rcees.ac.cn (Y.Y.); wtjiao@rcees.ac.cn (W.J.); 2 College of Resources and Environment, University of Chinese Academy of Sciences, Beijing 101408, China

**Keywords:** comprehensive evaluation method, contaminated soil, ex situ thermal desorption, environmental impact, resource utilization

## Abstract

Quantitative evaluation of different contaminated soil remediation technologies in multiple dimensions is beneficial for the optimization and comparative selection of technology. Ex situ thermal desorption is widely used in remediation of organic contaminated soil due to its excellent removal effect and short engineering period. In this study, a comprehensive evaluation method of soil remediation technology, covering 20 indicators in five dimensions, was developed. It includes the steps of constructing an indicator system, accounting for the indicator, normalization, determining weights by analytic hierarchy process, and comprehensive evaluation. Three ex situ thermal desorption technology—direct thermal desorption, indirect thermal desorption, and indirect thermal heap—in China were selected for the model validation. The results showed that the direct thermal desorption had the highest economic and social indicator scores of 0.068 and 0.028, respectively. The indirect thermal desorption had the highest technical and environmental indicator scores of 0.118 and 0.427, respectively. The indirect thermal heap had the highest resource indicator score of 0.175. With balanced performance in five dimensions, the indirect thermal desorption had the highest comprehensive score of 0.707, which is 1.6 and 1.4 times higher than the direct thermal desorption and indirect thermal heap, respectively. The comprehensive evaluation method analyzed and compared the characteristics of the ex situ thermal desorption technology from different perspectives, such as specific indicators, multiple dimensions, and single comprehensive values. It provided a novel evaluation approach for the sustainable development and application of soil remediation technology.

## 1. Introduction

Establishing a comprehensive and practical evaluation system is of critical importance to the sustainable development of technologies. Comprehensive evaluation refers to the use of a systematic and standardized method that includes simultaneous multiple indicators for evaluation. Comprehensive evaluation can analyze the whole process of technology implementation, and provide information for process optimization in terms of technological, economical, and social aspects [1,2,3]. Therefore, comprehensive evaluation is very important for process optimization of technology, and the comparison and selection among different technologies.

Contaminated soil remediation is an important issue in the environmental field [4]. In past decades, a variety of soil remediation technologies have been developed [5]. To evaluate different soil remediation technologies, one first needs to focus on the characteristic indicators such as efficiency, stability, and applicability. The technology consumes raw and auxiliary materials, and energy during implementation, resulting in the consumption of natural resources. At the same time, emissions from energy consumption or process physicochemical reactions can result in environmental impacts. The economic cost, benefit, and technical value of the technology are also important factors of concern to investors and decision makers. In addition, the implementation of such pollutant removal technologies can have certain social impacts, such as job opportunities for local residents, but also negative social effects, such as concerns from adjacent residents and potential impact on workers’ health. A comprehensive evaluation can avoid the transfer of technological loads between different dimensions.

Ex situ thermal desorption has become one of the most effective remediation technologies for organic contaminated soil [6,7,8,9]. Ex situ soil remediation usually is the second choice after in situ technology, which are more sustainable and less costly; thus, the effort to analyze the impact of the ex situ remediation processes is necessary. Since the 1980s, scholars from the United States, France, Canada, Argentina, South Korea, and other countries have carried out thermal desorption remediation research on a variety of organic contaminated soils [10]. In Europe, thermal desorption has also been widely used in engineering practice [6,11,12,13,14]. In America, among the 571 ex situ soil remediation projects carried out during 1982 to 2014, 77 used ex situ thermal desorption remediation technology, accounting for 13.5% of the total number of projects [14]. The independent research, and the development and application of the equipment for ex situ thermal desorption technology in China started late. The first patent on thermal desorption remediation technology was granted in 2009, and the first related article was published in 2011 [15,16]. As of 2017, a total of 23 ex situ thermal desorption remediation projects for contaminated sites have been carried out [15].

At present, the evaluation of carbon emission and environmental impact of ex situ thermal desorption technology has been carried out [17,18,19,20], but there are few literature reports on its quantitative evaluation at the levels of different dimensions, such as technical characteristics, resources, environment, economy, and society. A comprehensive study can provide a theoretical basis for the directional selection of ex situ thermal desorption technology in terms of specific indicators, and further provide scientific support for the overall development of ex situ thermal desorption technology. In this paper, multilevel comprehensive evaluation is carried out for direct and indirect ex situ thermal desorption technology, and its key influencing factors and advantageous indicators are determined through comparative analysis, which further reflects the importance of technology evaluation methods in selecting appropriate technology. The establishment of a comprehensive evaluation model is conducive to the optimization, improvement, and comparative selection of technology, and can provide a new analytical method for the quantitative comparison between different ex situ thermal desorption technologies.

## 2. Method and Data

### 2.1. Methodological Framework

**Figure 1:**
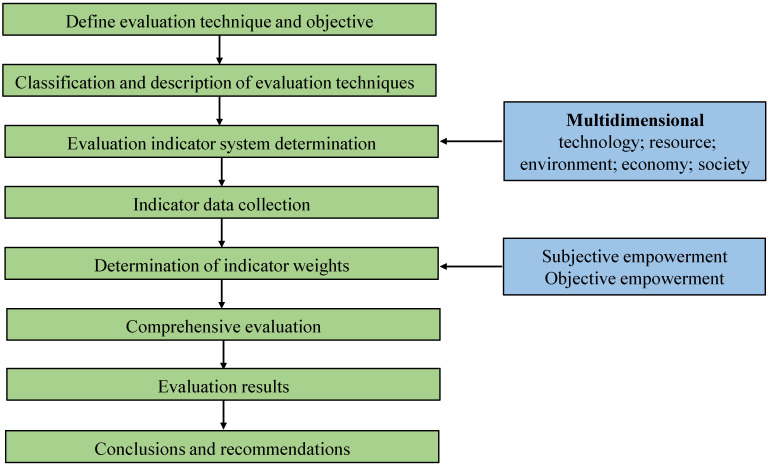
Comprehensive evaluation method framework.

A comprehensive evaluation method for ex situ thermal desorption technology was constructed in this study, and its framework is shown in Figure 1. The main steps of technology evaluation include: (1) determining the evaluation object and the technology involved in the evaluation; (2) describing the remediation technology; (3) determining the evaluation indicator set and collecting the evaluation indicator parameters; (4) determining the weight and quantification method of the evaluation indicator; (5) comprehensively analyzing and weighting each indicator, and calculating the score of each evaluated dimension; and (6) obtaining the comprehensive evaluation result.

### 2.2. Comprehensive Evaluation Indicator System

#### 2.2.1. Evaluation Indicators

To comprehensively evaluate the performance of ex situ thermal desorption technology, an evaluation indicator system was constructed by referring to a sustainable development indicator, a green development indicator, and an environmental pollution prevention and control technology evaluation method. It contains five dimensions, which are technology, resource, environment, economy, and society, and has a total of 20 indicators. The dimensions and the indicators are shown in Table 1.

(1) Technical indicators

In terms of technical indicators, characteristics of efficiency and operation are constructed. The efficiency indicators reflect the characteristics of the technology in pollutant removal and thermal efficiency; the operation indicators reflect whether the technology still has instability. The technical efficiency indicators select heat transfer efficiency and pollutant removal rate; the operational indicators select secondary pollutants, fault condition, and comprehensive energy consumption as secondary indicators.

(2) Resource indicators

The resource indicators reflect the demand for various material inputs in the process of technology implementation, which select raw material consumption, energy consumption, and water consumption as secondary indicators.

(3) Environmental indicators

Environmental indicators include two parts, the first part is the environmental impact during the implementation of technology, focusing on noise and peculiar smell, and the second part is the whole process environmental impact, in which we applied the life cycle assessment (LCA) method to calculate the typical environmental impact. This study selects global warming potential (GWP), eutrophication potential, acidification potential, and ozone layer depletion potential.

(4) Economic indicators

Economic indicators are designed to reveal the costs and benefits of technology, which select investment return period, direct benefits, and indirect benefits as secondary indicators.

(5) Social indicators

Social indicators reflect the basic social benefits and negative effects. The social benefits include the job opportunities and social income, while the negative effects primarily consider the concerns from the neighboring residents, i.e., “not in my back yard” (NIMBY).

#### 2.2.2. Quantification of Evaluation Indicators

Once the indicator system is defined, the indicators need to be quantified and normalized. The indicators are divided into qualitative and quantitative indicators; qualitative indicators are graded according to the severity, and quantitative indicators are calculated based on the definitions of the indicators.

The indicators, with different units and magnitudes, need to be normalized. The common methods of evaluation indicators are mainly the normalization method (quantitative indicators) and rank assignment method (qualitative indicators) [21]. These two methods are used in this study.

To ensure the accuracy of assessment, all quantitative indicators were normalized before calculating the weights, with the largest values selected as criterion 1 for positive indicators and the smallest values selected as criterion 1 for negative indicators, with all indicator values between 0 and 1.

### 2.3. Weight Determination Method

#### 2.3.1. Weighting Calculation

The determination of the weight coefficient can be performed by using the expert scoring method, target distance method, analytic hierarchy process (AHP), or entropy weight method [22,23]. The expert scoring method mainly collects experts’ opinions on the importance of relevant indicators according to the relevant knowledge mastered by experts in the opinion table, and summarizes the different opinions to reach common opinions. The target distance method is widely used in the environmental field and it mainly represents the severity of the environmental impact effect based on studying the gap between the current level and the target level [24]. In this paper, we choose the AHP, which is mainly used to solve the problem of decision making, with a combination of qualitative and quantitative method. The direct participation of decision makers ensures the consistency of the model thinking process, which can provide support for various fields with complex problems [25,26].

#### 2.3.2. Judgment Matrix Construction

The weight coefficients in this study were determined by the AHP. The importance scales of different indicators in this method and their meanings are shown in Table 2 [27].

Table 2 quantifies the relative importance of indicators in different dimensions. On this basis, the weight of each indicator can be calculated according to the root method or the sum product method. According to a number of expert opinions and literature reports [28], combinedwith judgment matrix construction, the importance scale of dimensions or indicators is determined, as shown in Table 3, Table 4, Table 5, Table 6, Table 7 and Table 8.

#### 2.3.3. Weighting Coefficient Determination

Based on the analysis above, this study uses the sum product method to calculate the weight coefficient, which can be divided into two steps:

First, the judgment matrix is normalized by column, and the rows are added and summed as follows:

$${\bar{W}}_{i}=\overset{n}{\underset{j=1}{\sum }}\frac{a_{ij}}{\sum_{i=1}^{n}a_{ij}}$$

Second, normalization is carried out, and the result is the weight coefficient of each environmental indicator, which can be obtained from the following formula:

$$W_{i}=\frac{{\bar{W}}_{i}}{\sum_{i=1}^{n}{\bar{W}}_{i}}$$

The weight coefficient results obtained are shown in Table 9.

#### 2.3.4. Consistency Test of Judgment Matrix

Inconsistent judgments may derive from the comparison matrix obtained by the two-by-two comparison method used in AHP. Therefore, a consistency test is required. Additionally, the consistency test mainly refers to the fact that when variable a is relative important to variable b, and variable b is relative important to variable c, then variable a must be more important than variable c.

The consistency indicator *CI* is

$$CI=\frac{\left(\lambda_{max}-n\right)}{\left(n-1\right)}$$

The formula for determining consistency is

$$CR=\frac{CI}{RI}<0.1$$

where *CR* is the consistency ratio. *RI* is the average random consistency indicator, and its value is shown in Table 10.

Based on the calculation and analysis of the formula above, the consistency test results obtained for the five dimensions are shown in Table 11.

As can be seen from the these tables, the consistency ratio of importance ranking of all indicators is less than 0.1, indicating that the ranking results have a satisfactory consistency and can be accepted [29].

### 2.4. Comprehensive Evaluation Methodology

The total score *S* can be obtained from the weighted average of the values of each dimension:

$$S=\sum_{i=1}^{p}D_{i}$$

where *S* is a single indicator of comprehensive evaluation and *D_i_* is the score of each indicator/dimension at different levels.

### 2.5. Data Source

The data obtained from the inquiry of remediation site staff, inspection of project reports, and test reports are used in this study, as shown in Table 12. The data of this study are divided into five categories. The first is the data of resource and energy consumption, and environment emissions, which mainly come from the statistical, recorded, and monitor data on the remediation site. The technical data mainly come from interviews of technicians at the remediation site, and most of the economic and social data come from the technology report. The data of NIMBY came from the survey and interview of nearby residents. In addition, the basic data of LCA designed in the environmental dimension mainly come from CAS RCEES 2020 developed by the Research Center for Eco-Environmental Sciences, Chinese Academy of Sciences. This database supports the publication of many related studies [21,30,31].

## 3. Case Study

### 3.1. Remediation Site and Technology Selection

**Figure 2:**
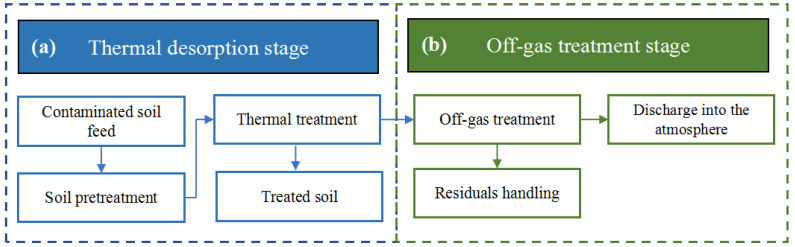
Basic process of a thermal desorption system.

Thermal desorption can be divided into two parts: the thermal desorption stage and the off-gas treatment stage, as shown in Figure 2. Ex situ thermal desorption technologies involve excavating and transporting contaminated soil from the original site where the pollution occurred to other sites for remediation. The principle is that through direct or indirect heating, the contaminated soil reaches a certain temperature, in which the organic pollutants are converted into a gas phase and volatilized into the desorption off-gas, and then completely removed by the gas treatment system, so as to obtain clean soil [32,33]. According to different contact modes between heat source and contaminated soil, ex situ thermal desorption technology can be divided into direct and indirect thermal desorption technology [5,34].

**Figure 3:**
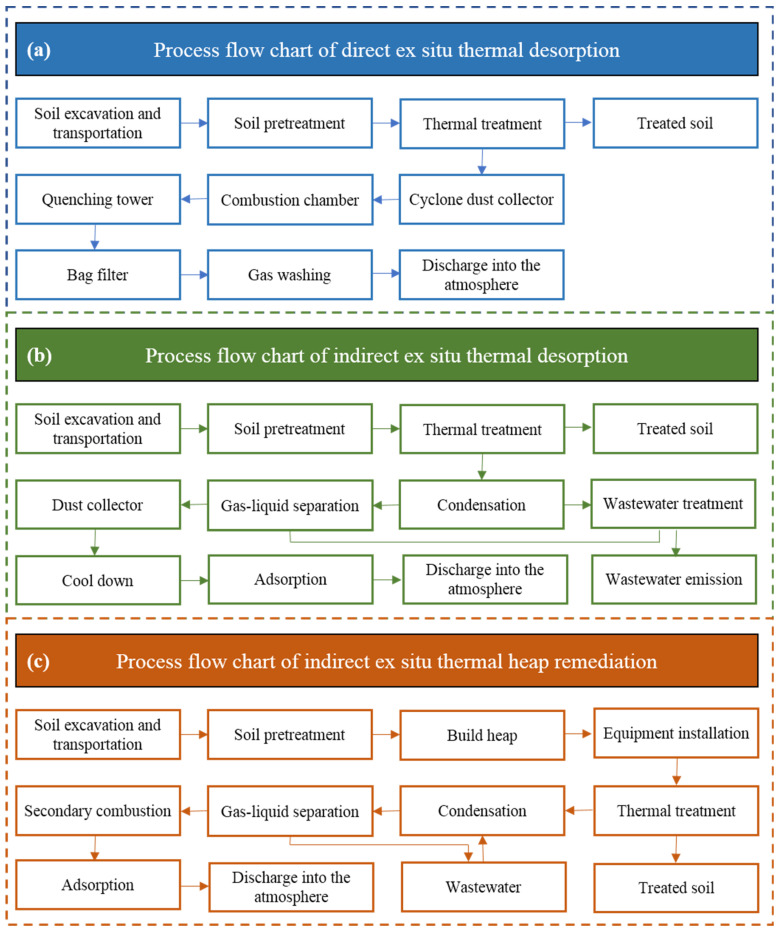
Flow chart for three ex situ thermal desorption technology.

In this study, ex situ thermal desorption technologies were selected at contaminated sites in three cities of China, and their process flow chart is shown in Figure 3.

Direct ex situ thermal desorption is adopted at the Tianjin contaminated site in China, with the process flow chart shown in Figure 3a. The soil is dehydrated, screened, and loaded in the pretreatment workshop. Rotary kilns use natural gas as raw material to heat the soil. The heated soil is humidified and cooled with water, and the off-gas generated in the treatment process is discharged into the atmosphere by cyclone dust collector, combustion chamber, quenching tower, bag filter, and gas washing. No waste water is generated during thermal desorption.

Indirect ex situ thermal desorption is adopted at the Liuzhou contaminated site in China, with the process flow chart shown in Figure 3b. The soil is dehydrated, screened, and loaded in the pretreatment workshop. Rotary kilns use natural gas as raw material to heat the soil. The heated soil is humidified and cooled with water, and the off-gas generated in the treatment process is discharged into the atmosphere by condensation, gas–liquid separation, dust collector, cool down, and adsorption. The wastewater generated is collected in the collection tank and pumped to the wastewater treatment equipment on-site before being discharged.

Indirect thermal heap remediation is adopted at the Linyi contaminated site in China, with the process flow chart shown in Figure 3c. The construction of thermal heap includes the following steps: pretreatment of soil, construction of the heap body, fuel system, heating and extraction system installation, and the auxiliary system installation. The off-gas generated in the treatment process is discharged into the atmosphere by condensation, gas–liquid separation, secondary combustion and adsorption, and the wastewater generated is collected and stored in a temporary storage system.

### 3.2. Results Analysis

The results obtained by this method can show the characteristics of technology and comparison from different angles: (1) Analyze the specific parameters and improvement hotspots of the technology from the specific indicator performance, such as the GWP, and emission sources that caused GWP. (2) Trace the improvement direction from different dimensions and show the balance characteristics of the technology between dimensions. For example, the poor performance of the resource dimension indicates that it has high demand for resources, energy, and raw and auxiliary materials. At the same time, we should comprehensively consider technology from different dimensions, not only pursue one dimension and ignore the other dimensions. For example, the performance of technical efficiency and operation process is well, but the environmental impact is high. (3) Promptly judge the comprehensive performance of different technologies under a single indicator of comprehensive evaluation.

**Figure 4:**
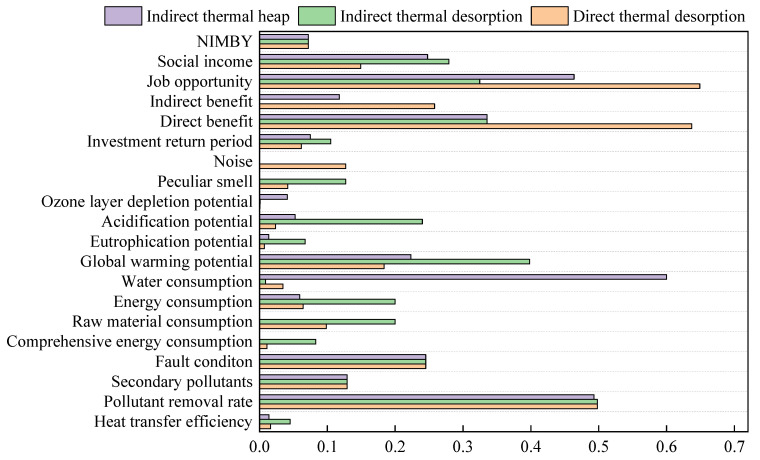
Secondary indicators of the three ex situ thermal desorption processes.

**Figure 5:**
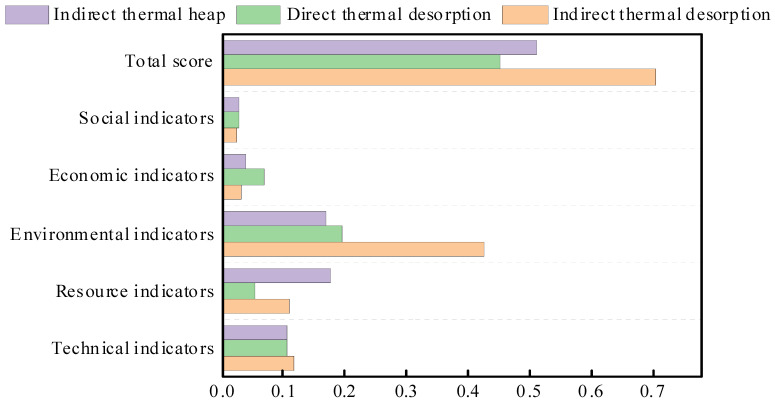
Comparison of primary indicators for the three ex situ thermal desorption processes.

**Figure 6:**
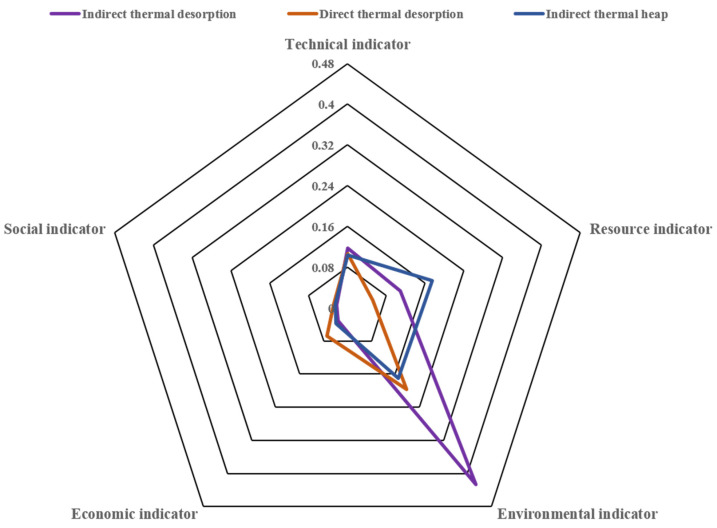
Comprehensive comparison radar map for the three ex situ thermal desorption technology.

Primary and secondary indicators of the three kinds of ex situ thermal desorption is demonstrated in Table 13. The comparison of secondary indicators of the three ex situ thermal desorption is shown in Figure 4, the comparison of primary indicators of the three ex situ thermal desorption is shown in Figure 5 and the comprehensive comparison radar chart is shown in Figure 6.

#### 3.2.1. Indicator Performance

In terms of technical indicators, the conclusion drawn from Table 13 shows that three kinds of ex situ thermal desorption have different advantages on five indicators. The scores of three technical indicators of the indirect thermal heap are lower than those of the direct thermal desorption and indirect thermal desorption, whereas the scores for heat transfer efficiency and comprehensive energy consumption of the indirect thermal desorption are higher than those of the direct thermal desorption and indirect thermal heap. Overall, the indirect thermal desorption has the best technical indicator.

The score of raw material and energy consumption of the indirect thermal desorption is higher than those of direct thermal desorption and indirect thermal heap, but the score of water consumption of the indirect thermal heap is much higher than that of direct thermal desorption and indirect thermal desorption. Overall, owing to less raw material consumption, the indirect thermal heap has the best resource indicators.

The scores for GWP, eutrophication potential, acidification potential, and peculiar smell indicators of the indirect thermal desorption are higher than those of the direct thermal desorption and indirect thermal heap, and the noise of the direct thermal desorption is higher than that of the indirect thermal desorption and indirect thermal heap. The best performance of the indirect thermal desorption in the GWP is due to the low energy consumption. Overall, the indirect thermal desorption has the best environmental indicators.

The score of direct and indirect benefit of the direct thermal desorption is higher than that of the indirect thermal heap, while the indirect thermal desorption has least indirect benefit. In general, the direct thermal desorption has the best economic indicator.

The direct thermal desorption has more job opportunities than that of the indirect thermal desorption and indirect thermal heap, and the indirect thermal desorption’s social income is higher than that of the direct thermal desorption and indirect thermal heap. In general, the direct thermal desorption has the best social indicators.

#### 3.2.2. Dimensional Analysis

It can be seen from Figure 4 and Figure 5 that the indirect thermal desorption has the highest environmental indicator score among the three ex situ thermal desorption technology. Specifically, the environmental indicator of indirect thermal desorption is 2.1 times and 2.5 times higher than that of direct thermal desorption and indirect thermal heap, respectively. The primary reason is that indirect thermal desorption has much higher scores in peculiar smell, acidification potential, and GWP. The direct thermal desorption has the highest economic indicator score, 2.2 times and 1.8 times higher than the indirect thermal desorption and indirect thermal heap, respectively, mainly in the indirect and direct benefits. The indirect thermal heap has the highest resource indicator score, which is 3.3 times and 1.6 times higher than the direct thermal desorption and indirect thermal desorption, respectively, mainly in water consumption.

Combining the weights of the five first-level indicators, the indirect thermal desorption has the highest total score, 1.5 times and 1.4 times higher than the direct thermal desorption and indirect thermal heap, respectively. In general, the overall scoring order of the three ex situ thermal desorption technology is as follows: indirect thermal desorption > indirect thermal heap > direct thermal desorption. In the comprehensive evaluation and analysis of this case, when selecting a remediation method for a contaminated site, in addition to considering the site’s own situation, it can also provide a technical basis for its key indicators, which is comparatively reference-valuable.

The information shown in Figure 6 suggests that the three ex situ thermal desorption processes have their own advantages in five dimensions: the indirect thermal desorption ranks highest in terms of environmental indicators and technical indicators, especially the environmental indicators. The environmental benefits of the indirect thermal desorption are far superior to the other two sites. In terms of both social indicators and economic indicators, the direct thermal desorption has a score that is higher than the other two technologies, and the economic indicators of the direct thermal desorption are more advantageous. The indirect thermal heap achieves the highest score in terms of resource indicators. During comparison of the ex situ thermal desorption, it is notable that although all of the three sites adopt ex situ thermal desorption technology, the target pollutants removed are different, therefore the types and concentrations of secondary pollutants produced are different.

## 4. Discussion

### 4.1. Indicator System

In order to construct a multidimensional indicator system for the evaluation of ex situ remediation technologies, this study developed a research framework, as shown in Figure 1. Different published indicator systems developed for other research purposes (e.g., green development) were referenced in this study. Indicators related to the evaluation of ex situ remediation technologies were selected from the relevant literature, and these indicators were employed in our study to form a system of indicators applicable to ex situ remediation technologies.

Technical indicators mainly consider the factors in the technical recommendation list. Resource indicators mainly cover the consumption of resources, energy, and materials. Environmental indicators draw on the LCA method of environmental impact assessment and focus on important factors such as GWP. The economic indicators mainly consider the cost, rationality, and benefit. The social dimension takes into account the two stakeholders, which are local communities and workers.

### 4.2. Methodological Applicability

In this study, three kinds of ex situ thermal desorption remediation technologies are selected to verify the credibility of the model. There are some studies on the environmental impact assessment of the technology, but all use the LCA method to evaluate the GWP or other environmental impacts. However, only GWP, an indicator or environmental impact dimension, cannot be used to evaluate the comprehensive sustainability of the technology [22]. Different from the above research, the evaluation model developed in this study covers five dimensions—technology, resources, environment, economy and society—which creates a more comprehensive evaluation.

In this study, the AHP and comprehensive evaluation are used to empower and aggregate the five dimensions of sustainability performance. The results show that indirect thermal desorption has the highest score of technical and environmental indicators, indirect thermal heap has the highest score of resource indicators, direct thermal desorption has the highest score of economic and social indicators. With the balanced performance of five dimensions, indirect thermal desorption has the highest comprehensive evaluation, which shows the importance of the comprehensive model.

The case study shows that the current comprehensive evaluation model developed in this study can be widely applied to various remediation technologies, and can reveal the characteristics of the technologies. Firstly, during the case study, the data are easy to collect and obtain, and the calculation of the indicators is straightforward. Secondly, in the process of weight determination and comprehensive evaluation, the methodology is mature and easy to operate. The results of the study reflect the characteristics of the technologies at three levels: specific indicators, dimensions, and single indicators of comprehensive. The analysis of different types of ex situ thermal desorption cases shows that the model can be well applied to the evaluation of this case. For other types of technologies, this model can also be used to obtain reliable results.

In addition, the process optimization conclusions of this study can also be applied to other technical cases. For example, reducing energy consumption and increasing the proportion of renewable energy, which can reduce resource and energy consumption, reduce environmental impact, improve technical performance, and reduce costs to a certain extent. Social impact is also a link that must be given attention in the use of remediation technology, such as improving employee welfare and reducing adverse effects such as NIMBY, which can promote the process optimization and market application of technology.

## 5. Conclusions

Comprehensive evaluation of different soil remediation technologies is of critical importance to the optimization and selection of proper technology. Comprehensive evaluation refers to the use of a systematic and standardized method for simultaneous evaluation of multiple indicators. It includes the steps of constructing an indicator system, accounting for the indicators, normalization, determining weights by AHP, and comprehensive evaluation.

In this study, a comprehensive evaluation method of soil remediation technology, covering 20 indicators in five dimensions, was developed. Three ex situ thermal desorption processes—direct thermal desorption, indirect thermal desorption, and indirect thermal heap—were selected for the method validation. The results showed that direct thermal desorption had the highest economic and social indicator scores. Indirect thermal desorption had the highest technical and environmental indicator scores. Indirect thermal heap had the highest resource indicator score. With balanced performance in five dimensions, the overall comprehensive score order of the three ex situ thermal desorption is indirect thermal desorption > indirect thermal heap > direct thermal desorption. Our evaluation system can provide a theoretical basis for the improvement and selection of ex situ thermal desorption remediation technology. Our study can also provide a novel evaluation approach for the sustainable development and application of soil remediation technology.

## Figures and Tables

**Table 1:** Comprehensive evaluation indicators of ex situ thermal desorption remediation technology.

Dimensions	Indicators	Units	Indicator Definition
Technical indicators	Heat transfer efficiency	%	Heat transfer rate per unit time
	Pollutant removal rate	%	Removal rate of target pollutants (removal rate to standard)
	Secondary pollutants	/	Whether to produce other secondary pollutants (produce exceed the standard, produce but not exceed, not produce)
	Fault condition	/	Whether it can operate stably and produce failure situations (no fault, minor fault, and serious fault affect the operation)
	Comprehensive energy consumption	MJ/t soil remediation	Energy consumption during operation
Resource indicators	Raw materials consumption	kg/t soil remediation	Whether to consume dehydrating agents, conditioning agents, odor inhibitors, etc.
	Energy consumption	kWh, m^3^, L ect./t soil remediation	Consumption of electricity, natural gas, gasoline, etc., from life cycle perspective
	Water consumption	m^3^/t soil remediation	Fresh water consumption
Environmental indicators	Global warming potential	kg CO_2_-Equiv./t soil remediation	Life cycle assessment methodology indicator
	Eutrophication potential	kg Phosphate-Equiv./t soil remediation	Life cycle assessment methodology indicator
	Acidification potential	kg SO_2_-Equiv./t soil remediation	Life cycle assessment methodology indicator
	Ozone layer depletion potential	kg R11-Equiv./t soil remediation	Life cycle assessment methodology indicator
	Peculiar smell	/	Peculiar smell during the implementation of technology
	Noise	decibel	Noise impact during implementation of technology
Economic indicators	Investment return period	Year	The number of years from the time the project starts production to the time when the full construction investment is recovered
	Direct benefit	Yuan (RMB)/t soil remediation	Net profit of remediation of unit contaminated soil
	Indirect benefit	Yuan (RMB)/t soil remediation	Disposal costs reduced by remediation of unit contaminated soil
Social indicators	Job opportunity	person/t soil remediation	Jobs created during the operation
	Social income	%	The income level of practitioners, the income per person per month/local average income
	“Not in my back yard” (NIMBY)	/	Residents or local units worry that remediation technology will bring many negative effects on health, environmental quality, and asset value

**Table 2:** Indicator importance scale.

Importance Scale *a_ij_*	Description	Importance Scale *a_ij_*	Description
1	Two factors have the same importance	9	*i* is more important than *j*
3	*i* is slightly more important than *j*	2,4,6,8	scale median
5	*i* is more important than *j*	reciprocal	*j* compared to *i*
7	*i* is extremely more important than *j*		

**Table 3:** Importance scale of different dimensional layers.

	Technology	Resources	Environment	Economy	Society
Technology	1	1/3	1/5	3	3
Resources	3	1	1/3	5	7
Environment	5	3	1	7	9
Economy	1/3	1/5	1/7	1	5
Society	1/3	1/7	1/9	1/5	1
Weight	0.118	0.265	0.513	0.071	0.033

**Table 4:** Importance scale of the technical indicator.

	Heat Transfer Efficiency	Pollutant Removal Rate	Secondary Pollutants	Failure Situation	Comprehensive Energy Consumption
Heat transfer efficiency	1	1/7	1/3	1/5	1/3
Pollutant removal rate	7	1	5	3	5
Secondary pollutants	3	1/5	1	1/3	3
Fault condition	5	1/3	3	1	3
Comprehensive energy consumption	3	1/5	1/3	1/3	1

**Table 5:** Importance scale of the resource indicator.

	Raw Materials Consumption	Energy Consumption	Water Consumption
Raw materials consumption	1	1	1/3
Energy consumption	1	1	1/3
Water consumption	3	3	1

**Table 6:** Importance scale of the environmental indicator.

	Greenhouse Effect	Eutrophication	Acidification Effect	Ozone Layer Destruction	Peculiar Smell	Noise
Global warming potential	1	5	3	7	3	3
Eutrophication potential	1/5	1	1/3	3	1/3	1/3
Acidification potential	1/3	3	1	5	3	3
Ozone layer depletion potential	1/7	1/3	1/5	1	1/3	1/3
Peculiar smell	1/3	3	1/3	3	1	1
Noise	1/3	3	1/3	3	1	1

**Table 7:** Importance scale of the economic indicator.

	Investment Return Period	Direct Benefit	Indirect Income
Investment return period	1	1/5	1/3
Direct benefit	5	1	3
Indirect benefit	3	1/3	1

**Table 8:** Importance scale of the social indicator.

	Job Opportunity	Social Income	Adjacent Effect
Job opportunity	1	3	7
Social income	1/3	1	5
NIMBY	1/7	1/5	1

**Table 9:** Weight coefficient.

Primary Indicators	Secondary Indicators	Secondary Weight	Primary Weight
Technical indicator	Heat transfer efficiency	0.045	0.118
	Pollutant removal rate	0.498	
	Secondary pollutants	0.129	
	Fault condition	0.245	
	Comprehensive energy consumption	0.083	
Resource indicator	Raw materials consumption	0.200	0.265
	Energy consumption	0.200	
	Water consumption	0.600	
Environmental indicator	Global warming potential	0.398	0.513
	Eutrophication potential	0.067	
	Acidification potential	0.240	
	Ozone layer depletion potential	0.041	
	Peculiar smell	0.127	
	Noise	0.127	
Economic indicator	Investment return period	0.105	0.071
	Direct benefit	0.637	
	Indirect benefit	0.258	
Social indicator	Job opportunity	0.649	0.033
	Social income	0.279	
	NIMBY	0.072	

**Table 10:** Average random consensus indicator.

Numerical Value *n*	1	2	3	4	5	6	7	8	9
*RI*	0.00	0.00	0.58	0.90	1.12	1.24	1.32	1.41	1.45

**Table 11:** Consistency ratio of five dimensions.

	Technical Indicator	Resource Indicator	Environmental Indicator	Economic Indicator	Social Indicator
*CR*	0.066	0	0.051	0.037	0.064

**Table 12:** Description of data types and sources.

Data Types	Data Sources
Energy consumption and material consumption	On-site research
Technical specifications, failure situation, and efficiency	Provided by on-site technicians
Economic cost input and benefits	On-site research and project reports
Social employment and salary	On-site research and project reports
NIMBY	Survey and interview
Full process environmental impact base data	China localized life cycle assessment database CAS RCEES

**Table 13:** Comparison of primary and secondary indicators for the three ex situ thermal desorption processes.

Dimensions	Indicators	Secondary Indicators			Primary Indicators		
		Direct Thermal Desorption	Indirect Thermal Desorption	Indirect Thermal Heap	Direct Thermal Desorption	Indirect Thermal Desorption	Indirect Thermal Heap
Technical indicators	Heat transfer efficiency	0.016	0.045	0.014	0.106	0.118	0.104
	Pollutant removal rate	0.498	0.498	0.493			
	Secondary pollutants	0.129	0.129	0.129			
	Fault condition	0.245	0.245	0.245			
	Comprehensive energy consumption	0.011	0.083	0.009			
Resource indicators	Raw material consumption	0.099	0.200	0.001	0.052	0.108	0.175
	Energy consumption	0.064	0.200	0.059			
	Water consumption	0.035	0.009	0.600			
Environmental indicators	Global warming potential	0.184	0.398	0.223	0.197	0.427	0.169
	Eutrophication potential	0.007	0.067	0.014			
	Acidification potential	0.024	0.240	0.052			
	Ozone layer depletion potential	0.0005	0.0009	0.041			
	Peculiar smell	0.042	0.127	0			
	Noise	0.127	0	0			
Economic indicators	Investment return period	0.062	0.105	0.075	0.068	0.031	0.037
	Direct benefit	0.637	0.335	0.335			
	Indirect benefit	0.258	0.068	0.118			
Social indicators	Job opportunity	0.649	0.325	0.464	0.028	0.022	0.026
	Social income	0.149	0.279	0.248			
	NIMBY	0.072	0.072	0.072			
Total score					0.452	0.707	0.511

## Data Availability

Not applicable.
